# Sestrin2: multifaceted functions, molecular basis, and its implications in liver diseases

**DOI:** 10.1038/s41419-023-05669-4

**Published:** 2023-02-25

**Authors:** Chunfeng Lu, Yiming Jiang, Wenxuan Xu, Xiaofeng Bao

**Affiliations:** 1 School of Pharmacy, Nantong University, 226001 Nantong, Jiangsu China; 2 School of Life Science and Technology, China Pharmaceutical University, 210009 Nanjing, Jiangsu China

**Keywords:** Liver cancer, Liver diseases

## Abstract

Sestrin2 (SESN2), a highly conserved stress-responsive protein, can be triggered by various noxious stimuli, such as hypoxia, DNA damage, oxidative stress, endoplasmic reticulum (ER) stress, and inflammation. Multiple transcription factors regulate SESN2 expression, including hypoxia-inducible factor 1 (HIF-1), p53, nuclear factor E2-related factor 2 (Nrf2), activating transcription factor 4 (ATF4), ATF6, etc. Upon induction, SESN2 generally leads to activation of adenosine monophosphate-activated protein kinase (AMPK) and inhibition of mechanistic target of rapamycin complex 1 (mTORC1). To maintain cellular homeostasis, SESN2 and its downstream molecules directly scavenge reactive oxygen species or indirectly influence the expression patterns of key genes associated with redox, macroautophagy, mitophagy, ER stress, apoptosis, protein synthesis, and inflammation. In liver diseases including acute liver injury, fatty liver diseases, hepatic fibrosis, and hepatocellular carcinoma (HCC), SESN2 is abnormally expressed and correlated with disease progression. In NAFLD, SESN2 helps with postponing disease progression through balancing glycolipid metabolism and macroautophagy (lipophagy), and rectifying oxidative damage and ER stress. During hepatic fibrosis, SESN2 represses HSCs activation and intrahepatic inflammation, hindering the occurrence and progress of fibrogenesis. However, the role of SESN2 in HCC is controversial due to its paradoxical pro-autophagic and anti-apoptotic effects. In conclusion, this review summarizes the biological functions of SESN2 in hypoxia, genotoxic stress, oxidative stress, ER stress, and inflammation, and specifically emphasizes the pathophysiological significance of SESN2 in liver diseases, aiming to providing a comprehensive understanding for SESN2 as a potential therapeutic target in liver diseases.

## FACTS

- SESN2 is a stress-responsive protein with a distinct molecular structure regulating mTORC1.
- SESN2 is involved in multiple pathophysiological events, such as hypoxia, genotoxic stress, oxidative stress, endoplasmic reticulum stress, inflammation, autophagy, and cell death.
- SESN2 exerts potent hepatoprotective effects against acute and chronic liver injuries.

## OPEN QUESTIONS

- Does SESN2 have the potential as a viable therapeutic target for liver diseases?
- The roles of SESN2 in liver cancers should be further explored.

## Introduction

**Fig. 1:**
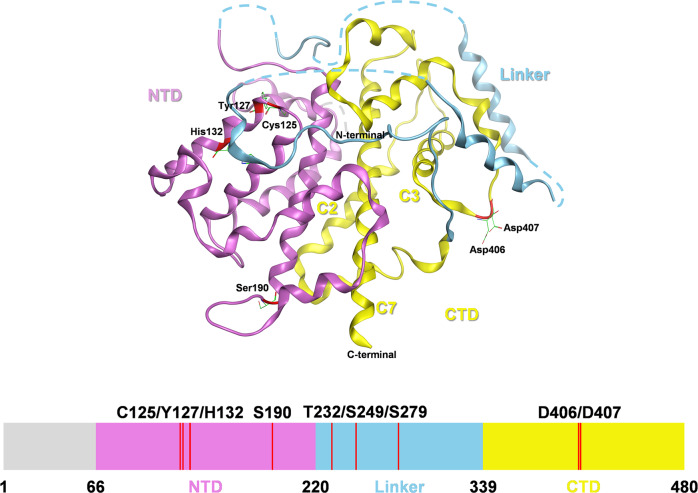
The crystal structure and functional domains of human SESN2. SESN2 is shown as a ribbon diagram with NTD, Linker, and CTD labeled as pink, blue, and yellow, respectively. The disordered regions (1–65, 222–232, 241–255, 272–280, 296–309) are labeled as dash lines. The locations of key residues (C125, Y127, H132, S190, T232, S249, S279, D406, and D407) are marked in red.

Sestrins (SESNs) belong to an evolutionarily conserved stress-responsive protein family existed in most vertebrates. The SESNs family comprises three members, SESN1, SESN2, and SESN3, among which SESN2 has been the most profoundly investigated [1]. SESN2, also nominated as Hi95, was originally identified inducible by prolonged hypoxia, DNA damage, and oxidative stress [2, 3]. Structurally, SESN2 is composed of two globin-like α-helix-only subdomains, N-terminal domain (NTD; residues 66–220) and C-terminal domain (CTD; residues 339–480), connected by a helix-loop-helix linker (residues 221–338). The NTD contains a homology region (residues 109–139) that corresponds to the helix-turn-helix oxidoreductase motif of an alkyl hydroperoxide reductase AhpD in *Mycobacterium tuberculosis*. The catalytic cysteine residue (Cys125) and the residues (Tyr127 and His132) mediating the proton delay system of AhpD are well-conserved within the NTD, enabling SESN2 to resemble AhpD in directly scavenging reactive oxygen species (ROS). The CTD contains a helix-loop structure but no helix-turn-helix motif or catalytic residues involved in AhpD oxidoreductase activity, implying that the CTD may not have the antioxidative activity. Aspartic acid residues Asp406 and Asp407 in the CTD may interact with GTPase-activating protein activity towards Rags 2 (GATOR2), liberating GATOR1 from GATOR2-mediated restriction [4]. GATOR1 binds to and inactivates Rag A/B, restricting lysosomal translocation and activation of mechanistic target of rapamycin complex 1 (mTORC1) [5]. Serine residue Ser190 in the NTD is also necessary for GATOR2 binding, suggesting that SESN2 may make multiple contacts with GATOR2 through both NTD and CTD [6]. There is a leucine-binding pocket at the intersection of helices C2, C3, and C7 in the CTD, which enables SESN2 to directly bind with leucine and act as a leucine sensor for transmitting leucine signal to activate mTORC1 [6–9]. The leucine pocket is in close proximity to the GATOR2 binding sites, which provides a possible mechanism for how leucine binding may cause SESN2 dissociation from GATOR2 that the changes in the leucine binding state alters the position of domains and affects the availability of GATOR2 binding sites [6]. Alternatively, leucine suppresses SESN2 phosphorylation at Thr232, Ser249, and Ser279, forcing SESN2 to dissociate from and activate GATOR2, leading to mTORC1 activation [10]. The unique molecular structure (Fig. 1) endows SESN2 with manifold roles in different biological processes.

As a classical stress-responsive protein, SESN2 can be induced in response to diverse stress conditions, such as hypoxia, genotoxic stress, and oxidative stress. In recent years, SESN2 has also been observed to be altered and impacted on endoplasmic reticulum (ER) stress and inflammation. Induced SESN2 may serve as a cellular defender against multiple detrimental stimuli and contribute to the recovery of organ homeostasis from diseases, especially liver diseases. In the liver, SESN2 displays additional functions including regulating glycolipid metabolism, HSCs activation, autophagy, cell survival and death.

In this review, we summarize the biological functions of SESN2 in distinct pathophysiological processes and particularly describe its association with liver diseases, aiming to promote profound understanding for the medical significance of SESN2.

## Diversified functions and regulations of SESN2 under stresses

### SESN2 in hypoxia

Hypoxia is a complex pathophysiological process occurring under diverse conditions. SESN2 was initially discovered as a prolonged hypoxia-induced molecule independent of p53 [2]. Later studies showed that SESN2 is transcriptionally activated by hypoxia-inducible factor-1 (HIF-1), a primary adaptive responsor to hypoxia [11, 12]. Induced SESN2 contributes to cellular self-protection by mitigating hypoxia-caused oxidative damage and cell death [2, 11, 13]. Another benefit of SESN2 to hypoxic injury is that SESN2 can deprive the induction of HIF-1 alpha subunit (HIF-1α) on vascular endothelial growth factor (VEGF) expression and brain-blood-barrier permeability, where may involve a mechanism that SESN2 facilitates HIF-1α degradation via enhancing the catalytic activity of prolyl hydroxylase (PH), an essential enzyme hydroxylating HIF-1α for ubiquitination [14]. Hence, SESN2 may be a preferable therapeutic target for hypoxia-related diseases.

### SESN2 in genotoxic stress

DNA damage can be caused by various endogenous or exogenous insults, including oncogenic mutations, oxidative stress, metabolic stress, etc. [15]. P53 is an important guardian of genome that can be activated under genotoxic and oxidative stress to promote genomic repair through induction of specific target genes. Upon DNA damage, SESN2 is induced in a p53-dependent manner [2]. SESN2 mediates p53-initiated mTORC1 inhibition, eliciting a metabolic checkpoint in response to genotoxic stress and executing the genomic guardianship [3, 16]. Serine/threonine kinase 3 (AKT3) signal is also involved in inducing SESN2 and then performing DNA repair [17]. This shows that SESN2 is a critical gatekeeper of genome.

### SESN2 in oxidative stress

Oxidative stress is triggered by redox imbalance after hyperactive oxidative system and/or hypoactive antioxidative defense. In response to oxidative stress, SESN2 can be transcriptionally activated by multiple transcription factors including nuclear factor E2-related factor 2 (Nrf2), CCAAT enhancer binding protein β (C/EBPβ), HIF-1, p53, activator protein-1 (AP-1), forkhead box protein O3 (FoxO3), nuclear factor-kappa B (NF-κB), and activating transcription factor 4 (ATF4) [18–25].

SESN2 architects cellular defense against redox imbalance generated by stimuli such as hydrogen peroxide [21], angiotensin II [26], methylglyoxal [27], etc., mainly via three patterns. Firstly, with the alkyl hydroperoxide reductase-like structure, SESN2 can directly function as an oxidoreductase to scavenge free radicals. However, the catalytically-crucial residue Cys125 within the NTD is surrounded by hydrophobic molecular surfaces, rendering SESN2 preferentially affinitive towards hydrophobic alkyl hydroperoxides rather than hydrogen peroxide. Also, its physiological substrates and reducing partners need clarification [4]. Secondly, SESN2 has physical-biological interactions with multiple redox regulators, among which the interaction between SESN2 and Nrf2-antioxidant-response element (ARE) antioxidant system has been the best studied [28–30]. Nrf2 is a core transcriptional regulator of antioxidant systems based on Mafs-mediated heterodimerization and ARE binding machinery. SESN2 but not SESN1 or SESN3 is exclusively induced at both transcriptional and translational levels by Nrf2 agonists and deprived when Nrf2 deletion. In silico and laboratory analyses displayed an ARE sequence (-550 to -539 bp) in the 5’ upstream region of *SESN2* gene promoter for Nrf2 binding [31]. Alternatively, induced SESN2 contributes to Nrf2 expression and nuclear translocation, amplifying the transcription of ARE-targeted antioxidant genes, including heme-oxygenase-1 (HO-1) and NAD(P)H: quinone reductase 1 (NQO1) [28].

**Fig. 2:**
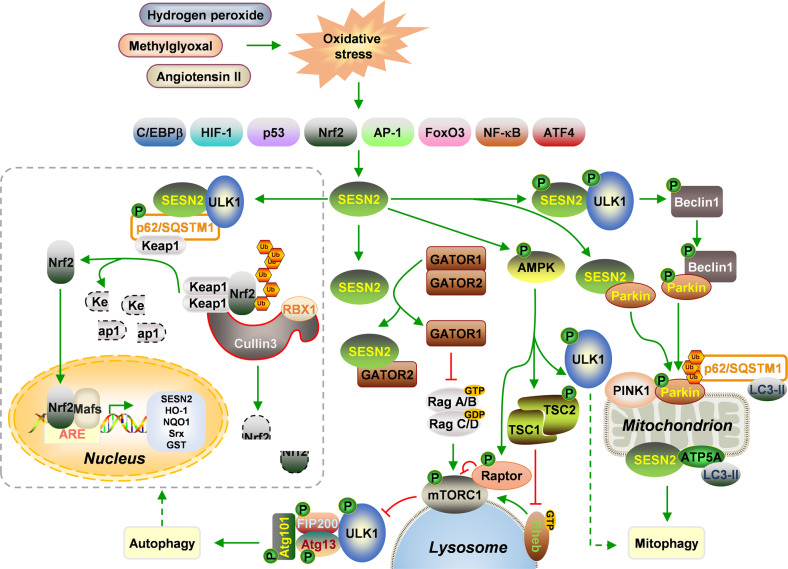
SESN2-regulated molecular network upon oxidative stress. SESN2 is up-regulated during oxidative stress induced by hydrogen peroxide, methylglyoxal, angiotensin II, etc., which is facilitated by transcription factors including C/EBPβ, HIF-1, p53, Nrf2, AP-1, FoxO3, NF-κB, and ATF4. (i) SESN2 restrains mTORC1 activity through directly binding to GATOR2. GATOR1 is released from GATOR1/2 complex and inactivates Rag A/B via promoting GTP hydrolysis, which prevents mTORC1 binding and recruitment to lysosome. ULK1 is dissociated, then auto-phosphorylates, phosphorylates the complex components including Atg13, FIP200, and Atg101, and promotes autophagy. SESN2 also indirectly inactivates mTORC1 in an AMPK-dependent way. SESN2 promotes AMPK activation and Raptor phosphorylation to inhibit mTORC1 activation. Alternatively, AMPK activation leads to TSC2 phosphorylation and inhibits GTP binding to Rheb, which halts mTORC1 activation. (ii) SESN2-ULK1 interaction promotes the autophagic degradation of Keap1 in a p62/SQSTM1-dependent manner, and accelerates Nrf2 nuclear translocation, which contributes to the formation of Nrf2/Mafs/ARE complex and the expression of downstream antioxidant genes including SESN2. (iii) SESN2 interacts with ULK1 and phosphorylates Beclin1, which anchors Parkin before its location to mitochondria, reinforces PINK1-Parkin interaction, and initiates mitophagy. SESN2 also interacts with Parkin, facilitating its mitochondrial translocation and mitophagy. Alternatively, SESN2 colocalizes with ATP5A on the outer mitochondrial membrane where ATP5A attaches LC3 directly to trigger mitophagy.

Thirdly, SESN2 can motivate macroautophagy (hereafter autophagy) machinery to clear defective proteins and organelles and recover redox balance, which is mainly achieved by inhibiting mTORC1, a pivotal checkpoint for autophagy, via both adenosine monophosphate-activated protein kinase (AMPK)-dependent and AMPK-independent mechanisms [32–35]. For AMPK-dependent mechanism, SESN2 increases the transcription of AMPKα1, AMPKβ1, and AMPKγ1 subunits, facilitates the formation of AMPKα1β1γ1 heterotrimer, and evokes AMPK activity via liver kinase B1 (LKB1)-catalyzed AMPKα1 phosphorylation at Thr127 [36]. AMPK phosphorylates tuberous sclerosis 2 (TSC2), the GTPase-activating protein (GAP) of the Ras homolog enriched in brain (Rheb). TSC2 facilitates the hydrolysis of Rheb-bound GTP and converts it to inactivated GDP-bound form, hindering Rheb interaction with the catalytic domain of mTOR and mTORC1 phosphorylation [3]. AMPK also phosphorylates the critical mTORC1 binding subunit regulatory associated protein of mTOR (Raptor) at two highly conserved serines, Ser722 and Ser792, and induces their direct binding to 14-3-3 protein, restricting the kinase activity of mTORC1 towards its downstream substrates [37]. mTORC1 when its catalytic activity is blocked unfreezes Unc-51-like protein kinase 1 (ULK1), which then forms an active complex via autophosphorylation and phosphorylation of autophagy-related protein 13 (Atg13), focal adhesion kinase interacting protein of 200 kD (FIP200), and Atg101, and activates autophagy [38]. ULK1 can also be phosphorylated by AMPK at multiple active residues and directly initiates autophagy [39]. In a second way independent of AMPK, SESN2 physically interacts with GATOR2 and releases GATOR1 acting as a GAP for Rag A/B, limiting mTORC1 translocation to lysosomal surface where to be activated by Rheb and provoking autophagy [40]. Physiological and pharmacological induction of SESN2 can contribute to autophagy marker light chain 3 (LC3)-II expression and autophagosome formation, suppressing mitochondrial dysfunction and oxidative stress [21, 41]. A possible mechanism for SESN2-regulated autophagy to ease oxidative stress may be that SESN2 physically associates with ULK1 and autophagic cargo receptor p62/sequestosome-1 (SQSTM1) to form a complex, facilitating p62/SQSTM1 phosphorylation at Ser403 and autophagic degradation of p62/SQSTM1 and its substrates [42], such as Kelch-like ECH-associated protein 1 (Keap1), a Nrf2 suppressor that can exclusively bind to the evolutionarily conserved N-terminal Neh2 regulatory domain of Nrf2 and facilitate its ubiquitylation and degradation in cytoplasm with the collaboration of Cullin3 and ring-box 1 (RBX1) [43, 44]. The autophagic degradation of Keap1 can promote the expression of Nrf2 downstream genes, including sulfiredoxin (Srx), glutathione-S-transferase (GST), and NQO1 [45]. More specifically, SESN2 can activate mitophagy, a mitochondrion-selective autophagic machinery, to remove damaged mitochondria for restoring redox homeostasis. Parkin is the predominant E3 ubiquitin ligase that can be recruited to mitochondria and phosphorylated by PTEN-induced kinase 1 (PINK1) upon mitochondrial damage. Then, Parkin integrates with ubiquitin and ubiquitylates substrates on mitochondrial outer membrane for recognition by autophagic cargo receptors and mitophagy formation. SESN2 amplifies PINK1/Parkin-mediated mitophagy by two main manners. On one hand, SESN2 interacts with ULK1 to phosphorylate Beclin1 at Ser14, promoting Beclin1 to bind to and phosphorylate Parkin and helping Parkin translocate to mitochondria [46]. Moreover, SESN2 can directly interact with Parkin, reinforcing the mitochondrial accumulation of Parkin [47]. On the other hand, SESN2 can facilitate the perinuclear-clustering of mitochondria by mediating p62/SQSTM1 aggregation and its binding to lysine 63 (K63)-linked ubiquitin on mitochondrial surface [48, 49]. There is another possible mode for SESN2 regulation on mitophagy that SESN2 can directly interact with mitochondrial alpha-subunit of F1-ATP synthase (ATP5A) through the CTD, attracting LC3-coated autolysosomes to locate ROS-damaged mitochondria for degradation [50]. There also forms a loop between ULK1 and SESN2 that SESN2 can be phosphorylated by ULK1 at Ser73 and Ser254, which is required for mitochondrial fusion with autophagosomes [50]. The multicomponent redox regulatory network centering on SESN2 is shown in Fig. 2.

### SESN2 in ER stress

Prolonged and unresolved ER stress are closely related to homeostasis disequilibrium and cell death. ER stress can activate three unfolded protein response (UPR) branches to orchestrate the recovery of ER function, including protein kinase RNA-like ER kinase (PERK)-eukaryotic translation initiation factor 2α (eIF2α)-ATF4 branch, inositol-requiring enzyme 1 alpha (IRE1α)-X-box binding protein 1 (XBP1) branch, and ATF6 branch [51, 52]. PERK-eIF2α branch can reduce protein synthesis. Paradoxically, preferential translation of ATF4 binds to C/EBP homologous protein (CHOP), a pro-apoptotic transcript, aggravates protein synthesis, ATP depletion, oxidative stress, and cell death [53, 54]. IRE1α-XBP1 and ATF6 branches can up-regulate the transcription of XBP1, glucose regulated protein 78 (GRP78), and GRP94, accelerating misfolded protein degradation and accurate protein folding [55, 56]. All three branches contribute to ER stress-mediated SESN2 induction, but the pathways involved vary among different stress inducers. ATF4 mediates both thapsigargin (Tg)- and brefeldin A (BFA)-induced SESN2 transactivation possibly by binding the site (-221 to -228 bp) within the upstream region of SESN2 transcription start site [57, 58]. XBP1 also transmits the activation signal of Tg to SESN2, but the regulatory pattern requires further verification [57]. ATF6 mainly governs tunicamycin (Tm)-triggered SESN2 enhancement, which can be implemented by being affinitive to UPR-element-like element 1 (UPRE-LE1) (-549 to -544 bp) and UPRE-LE6 (-235 to -230 bp) in the proximal half region of *SESN2* gene promoter [59].

**Fig. 3:**
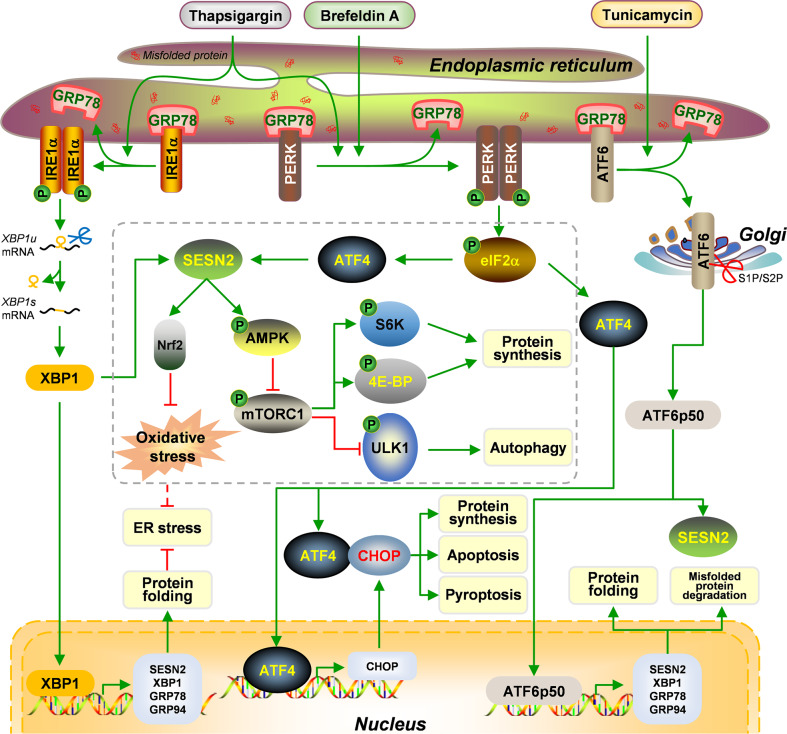
The regulatory mechanism of SESN2 during ER stress. ER stress agonists, thapsigargin, brefeldin A, and tunicamycin, can excite ER stress via inducing GRP78 dissociation and liberation of IRE1α, PERK, and ATF6. IRE1α phosphorylates and forms a dimer, cleaving XBP1 mRNA into an active form XBP1s. ATF6 when liberated translocates into Golgi and is lysed to ATF6p50 by S1P/S2P. Both XBP1 and ATF6p50 as transcription factors can up-regulate the transcription of downstream genes including XBP1, GRP78, GRP94, and SESN2, which promotes protein folding and misfolded protein degradation, and relieves ER stress. Induced SESN2 can arrest protein synthesis and enhance autophagy via AMPK-mTORC1 pathway and reduces oxidative damage via Nrf2. PERK phosphorylates and dimerizes, phosphorylating downstream elF2α and subsequently promoting the transcriptional activity of ATF4. ATF4 can initiate the transcription of CHOP, enhancing protein synthesis, apoptosis, and pyroptosis. ATF4 can also induce SESN2 expression.

Inducible SESN2 functions as a feedback modulator for ER stress and manipulates cell fates. SESN2 contributes to cell survival under Tg and BFA treatment, which is especially obvious at the early stimulation phase, indicating that progressive auto-activated SESN2 can mitigate mild ER stress [57]. Several studies have reached a consensus that SESN2 links ER stress to AMPK-mTORC1 signaling [60–65]. SESN2 deficiency augments ER stress-related signaling including PERK and IRE1α, which is associated with AMPK inactivation [61]. SESN2 knockdown also maintains PERK-eIF2α-CHOP signal transduction, leading to mTOR activation [66]. SESN2-mediated mTORC1 inhibition recovers ER homeostasis by two ways. One is that lack of mTOR-catalyzed phosphorylation of downstream molecules [ribosomal protein S6 kinase (S6K) and eIF4E-binding protein (4E-BP)] interrupts protein synthesis [60, 65]. The other one is that mTORC1 suppression initiates autophagy to eliminate misfolded proteins [63, 66, 67]. Cell death as a consequence of unresolved ER stress is also impressed by SESN2. SESN2 expression lessens ER stress-related apoptosis of dendritic cells (DCs), endothelial cells, and trophoblast cells [68–70]. SESN2 deficiency augments PERK-ATF4-CHOP signaling to induce NACHT, LRR, and PYD domains-containing protein 3 (NLRP3)/apoptosis-associated speck-like protein containing CARD (ASC)/Caspase-1-dependent cell pyroptosis [71, 72]. SESN2 can also defend against ER stress-associated non-canonical necroptotic death [73]. Furthermore, SESN2 can convalesce intracellular redox homeostasis to recover ER quality and function by enhancing the transcriptional activity of Nrf2 [74]. The modes of SESN2 to recover ER homeostasis is summarized in Fig. 3.

### SESN2 in inflammation

Inflammation generally involves both immune cells and non-immune cells. SESN2 is expressed in diverse immune cells, especially in macrophages and monocytes that are essential for innate immune response [75]. In bone marrow-derived mononuclear macrophages (BMDMs), NO and hypoxia transcriptionally activate SESN2 in a HIF-1α-dependent manner to resist cellular oxidative damage [20]. HIF-1α also mediates globular adiponectin (gAcrp)-induced SESN2 expression and anti-inflammatory response, which is under the regulation of extracellular regulated protein kinase (ERK)/phosphoinositide 3-kinase (PI3K) signaling [76]. Lipopolysaccharide (LPS) and other toll-like receptor (TLR) ligands (e.g. polyI:C and peptidoglycan) activate PI3K and p38 MAPK signals by conjugating with TLR, prompting AP-1 and Nrf2 induction on the transcription and translation of SESN2 [22, 77]. Inducible nitric oxide synthase (iNOS)-mediated NO production is also implicated in LPS-stimulated SESN2 elevation [48]. SESN2 can restrain p38 MAPK and c-Jun N-terminal kinase (JNK) phosphorylation to inhibit the DNA-binding activity of AP-1 and the activity of nicotinamide adenine dinucleotide phosphate (NADPH) oxidase, limiting release of pro-inflammatory cytokines [e.g. tumor necrosis factor alpha (TNF-α), IL-6, and IL-1β], ROS production, and cell death [77]. Oxidized low-density lipoprotein (OxLDL) elicits SESN2 expression by JNK/c-Jun signaling pathway to decay ROS generation and apoptosis in macrophages [78]. Furthermore, in a model of myocardial infarction, SESN2 is up-regulated in both pro-inflammatory M1 and anti-inflammatory M2 type cardiac macrophages, and SESN2 suppresses inflammatory response of M1 macrophages via inhibiting mTORC1 signaling and enhances M2 type macrophage polarization [79]. In cochlear tissues, SESN2 loss occurs with age and accelerates age-related sensory cell degeneration, which is correlated with overproduction of pro-inflammatory cytokines including TNF, chemokine (c-c motif) ligand 2 (CCL2/MCP-1), CCL3, CCL4, and IL-1β in cochlear macrophages [80]. In a model of acute cerebral ischemic stroke, ectopic SESN2 expression promotes the shifting of brain-resident macrophage/microglia from M1 to M2 phenotype and alleviates neuroinflammation by inhibiting mTOR pathway and restoring autophagic flux [81].

In monocytes, human acute monocytic leukemia cell line THP-1 cells for example, SESN2 is induced by LPS dose- and time-dependently, establishing a compensatory mechanism under p38 MAPK and PI3K activation by augmenting AMPK phosphorylation, decreasing NF-κB phosphorylation, and reducing secretion of pro-inflammatory cytokines (TNF-α, CCL2/MCP-1, and IL-6) [22, 61]. A later study supplemented that both high glucose and OxLDL can mediate monocyte polarization, which is characterized by increased M1 markers like iNOS, IL-6, TNF-α, etc. and decreased M2 markers like TGF-β, IL-10, etc., and monocyte adhesion to endothelial cells via SESN2-AMPK-mTOR nexus [82].

**Fig. 4:**
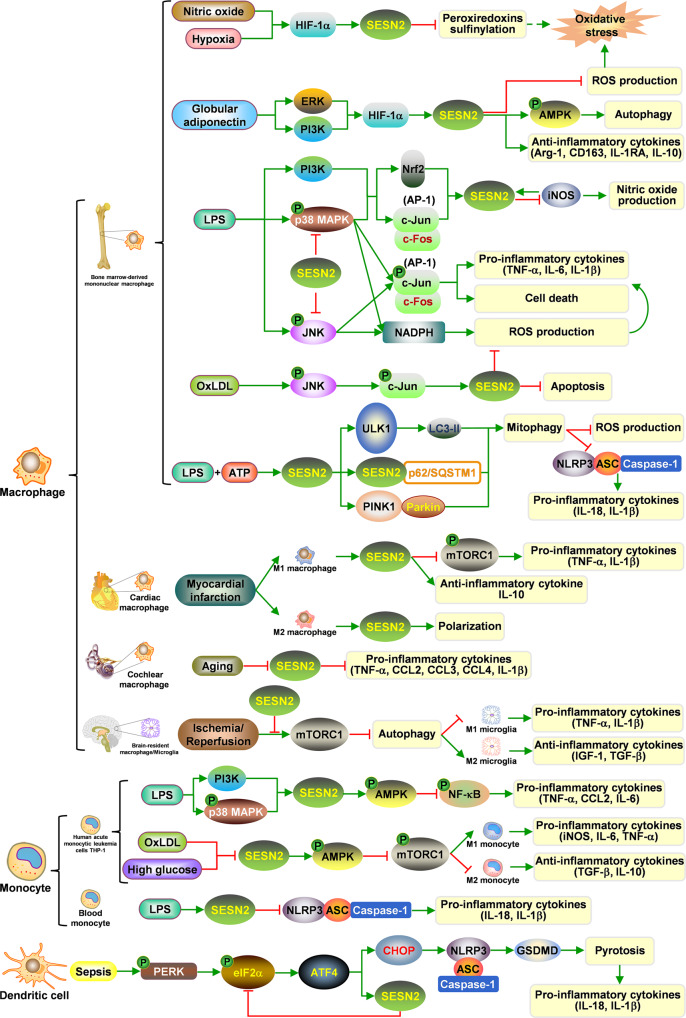
The roles of SESN2 in inflammatory response. SESN2 is expressed in immune cells including macrophages, monocytes, and dendritic cells, and architects cellular responses to inflammatory insults. SESN2 when induced by stimuli can exert robust anti-inflammatory function, which is associated with scavenging ROS, promoting autophagy, reducing inflammasome, and diminishing cell death.

NLRP3 is predominantly expressed in immune cells from the myeloid lineage, such as macrophages, monocytes, and DCs [83]. In *Sesn2*-knockout BMDMs, mitophagy is deficient but NLRP3 inflammasome is hyperactivated when primed with LPS and ATP. Mechanistically, SESN2 enhances p62/SQSTM1 aggregation to K63-ubiquitinated mitochondria. Synchronously, SESN2 facilitates ULK1-mediated initiation of autophagic machinery and launches the degradation of primed mitochondria, which diminishes mitochondrial ROS and cytosolic oxidized mitochondrial DNA generation, and suppresses prolonged NLRP3 inflammasome activation and inflammatory cytokine release (IL-1β and IL-18) [48, 84]. In an acute lung injury model, SESN2 suppresses NLRP3 activation and pyroptosis in alveolar macrophages via promoting PINK/Parkin-mediated mitophagy [49]. In sepsis models, SESN2 levels in blood monocytes negatively correlate with serum IL-1β and IL-18 levels and disease progression [48]. SESN2 also reduces gasdermin D (GSDMD)-dependent pyroptosis of splenic DCs in the context of sepsis via inhibiting PERK-ATF4-CHOP signaling-triggered NLRP3/ASC pyroptosome formation and Caspase-1 activation [71]. Collectively, SESN2 has pleiotropic functions in immune cells and exert robust anti-inflammatory activities (Fig. 4).

## Dynamics and control of SESN2 in liver diseases and implications

### Acute liver injury (ALI)

ALI is pathologically featured by extensive hepatocyte death and hypohepatia, which is generally caused by virus infection, drug abuse, hepatectomy, etc. Several studies collaboratively confirmed that preventative SESN2 overexpression can suppress galactosamine/LPS or acetaminophen-induced acute hepatocyte apoptosis and serum cytokine elevation, which is attributed to the antioxidative property of SESN2 [22, 77, 85]. Pharmacological induction of SESN2 by oleanolic acid also prevents hepatic ischemia reperfusion injury [86]. These findings uncover the potential of SESN2 regulation in the prevention of liver-related conditions.

### Fatty liver diseases

#### Non-alcoholic fatty liver disease (NAFLD)

NAFLD is one of the most common chronic liver diseases with a soaring worldwide prevalence, which generally starts from simple steatosis and progresses to steatohepatitis, hepatic cirrhosis, and/or hepatobiliary malignancies such as hepatocellular carcinoma (HCC) [87].

##### Glucose and lipid metabolism

Excessive fat deposition in hepatocytes, also mentioned as hepatocyte steatosis, is the most remarkable pathological manifestation of NAFLD, where obesity and insulin resistance are major risk factors [88]. SESN2 is the only isoform among three family members that is inducible by saturated fatty acids in hepatoma cell line HepG2 cells. However, under chronic NAFLD conditions, SESN2 gene expression in both human and murine livers is decreased [89]. *Sesn2* knockout aggravates glucose intolerance, insulin resistance, hepatocyte apoptosis, macrophage infiltration, and hepatic stellate cells (HSCs) activation in wild-type C57BL/6 J mice fed with high-fat diet (HFD) or in *Lep*^*ob/ob*^ mice, which involves AMPK inhibition and mTORC1-S6K activation; Ectopic SESN2 reconstitution can rescue SESN2-deficient mice from HFD-caused liver damage [60, 90]. Kowalsky et al. further elaborated that adenovirus-mediated systematic SESN2 overexpression decreases the expression of gluconeogenic and lipogenic genes in the liver, including acetyl-CoA carboxylase alpha (ACACA), ACACB, and fatty acid synthase (FASN), resulting in lower basal and insulin-reduced blood glucose levels, and hepatic lipid accumulation under HFD condition. Molecularly, they found that SESN2 induces AKT activation, an essential signal molecule responsible for glucose and lipid regulation, via mTORC2 but not mTORC1 or AMPK. SESN2 indirectly binds to mTORC2 relying on SESN2-GATOR2-mTORC2 interaction via WD repeat domain 24 (WDR24) and WDR59, which then facilitates AKT S473 phosphorylation. SESN2 also directly binds to the pleckstrin homology domain of AKT and induces AKT translocation to the plasma membrane. PI3K is also involved in the activation of AKT by SESN2 [91]. The regulatory action of SESN2 on hepatic lipogenesis is also relevant to liver X receptor alpha (LXRα), a transcription factor controlling *de novo* fatty acid synthesis. SESN2 induced by resveratrol represses LXRα-retinoid X receptor alpha (RXRα) DNA-binding activity and restricts the expression of lipogenic gene sterol regulatory element-binding protein-1c (SREBP-1c) and its target genes, including ACC, FASN, and stearoyl-CoA desaturase-1 (SCD1) [92, 93].

Accumulative evidence has shown that autophagy machine is impaired during the onset of NAFLD [94]. SESN2-deficient murine livers under HFD exposure show more and larger lipid droplets and lesser colocalization of lipid droplets and autophagic vesicles under transmission electron microscope, which implies deficient autophagic degradation of lipid droplets (as known as lipophagy) [90]. Notably, SESN2 induced by carbon monoxide restores autophagy in mouse hepatocytes AML12 cells and murine livers under methionine/choline deficiency (MCD) conditions via AMPK-mTORC1 axis, reducing triglyceride accumulation and hepatocyte damage [95]. These suggest that autophagy maintenance or enhancement may be a strategy for clearing excessive lipid droplets and ameliorating steatosis.

##### Oxidative stress and ER stress

Oxidative stress is another hallmark of NAFLD. Excessive free radicals can attack unsaturated fatty acids on biological membranes, inducing lipid peroxidation, destroying membrane structure, and leading to cell damage [96]. SESN2 is an important endogenous defender with prominent antioxidant capacity. Bae et al. found that SESN2 enhances p62/SQSTM1-mediated autophagic degradation of Keap1 and facilitates Nrf2 release and activation, thereby alleviating oxidative liver damage [45]. Han et al. further verified that pharmacological induction of SESN2 by liraglutide promotes the transduction of Nrf2/HO-1 pathway and initiates the translation of downstream targets, including catalase (CAT), NQO1, and glutamate cysteine ligase modifier subunit (GCLM), in livers of HFD mice, which contributes to the recovery of redox balance [97].

ER stress is also involved in the pathological mechanism of NAFLD. HFD evokes hepatic ER stress, characterized by increased expression of GRP78, ATF6, IRE1α, and eIF2α [60, 98]. Park et al. found that SESN2 is up-regulated by HFD via PERK-eIF2α-c/EBPβ signaling, which then regulates AMPK-mTORC1 axis to impede protein synthesis, relieve ER stress, and balance liver metabolism during obesity [60]. Jegal et al. linked SESN2 with oxidative stress and ER stress and confirmed the role of Nrf2-induced SESN2 in relieving Tm-induced ER stress-related liver injury [99].

#### Alcoholic fatty liver disease (AFLD)

AFLD, which is caused by chronic alcohol binge, is another major branch of fatty liver diseases. The disease spectrum of AFLD is similar to NAFLD and AFLD shares a large set of common pathological manifestations with NAFLD, such as hepatosteatosis, oxidative stress, ER stress, autophagy malfunction, etc. This suggests that the cytoprotective function and mechanism of SESN2 in AFLD may be analogous to that in NAFLD but still awaits further verification. More recently, Zhou et al. discovered that SESN2 is declined in murine livers after chronic alcohol exposure for 4 weeks and in human hepatocyte HL-7702 cells after 24-h alcohol stimulation. Pharmacological induction of SESN2 by pterostilbene significantly improves AFLD, featured by decreased serum aspartate aminotransferase (AST) and alanine aminotransferase (ALT) activity and reduced intrahepatic CD45^+^ leukocyte and F4/80^+^ macrophage/Kupffer cell infiltration. Mechanistically, pharmacologically-forced SESN2 expression promotes autophagic machinery and selective degradation of cellular communication network factor 1 (CCN1) via p62/SQSTM1, then mitigating hepatocyte senescence and senescence-associated secretory phenotype under alcohol exposure [100].

Together, SESN2 confers hepatocyte protection during fatty liver diseases, which is probably associated with regulation on glycolipid metabolism, oxidative stress, ER stress, autophagy, senescence, etc. (Table 1). Pharmacological induction and genetical reconstitution of SESN2 may be medically favorable for the improvement of fatty liver diseases.

**Table 1:** Roles of SESN2 in fatty liver diseases.

Disease type	Animal model	Intervention	Manifestations							Overall outcome	Reference
			Weight gain	Triglyceride level	Expression of lipogenic genes	Insulin resistance	Oxidative stress/ER stress/Inflammation/Apoptosis	Autophagy	Signaling		
NAFLD	Mice fasted for 16 h and refed with a high-carbohydrate, fat-free diet for 16 h	SESN2 knockout	No data	No data	No data	No data	Increased oxidative stress and apoptosis	Decreased	SESN2/p62/Keap1/Nrf2	SESN2 deprivation aggravates liver damage inflicted by acute lipogenic stimulation.	[45]
	(1) Mice fed with a HFD for 4 months; (2) 4-month-old Lep^ob/ob^ mice fed with a LFD	SESN2 knockout	No data	Increased	Modestly increased	Increased	Enhanced ER stress, inflammation, and apoptosis	No data	SESN2/AMPK/mTORC1	SESN2 knockout mice exhibit obvious hepatosteatosis and liver damage with fibrosis tendency.	[60]
	Mice fed with a WD for 8 weeks	SESN2 knockout	Modestly increased	Increased	Increased	No data	Increased oxidative stress, ER stress, inflammation, and apoptosis	No data	SESN2/mTORC1/JNK	SESN12 knockout and SESN1/2/3 triple knockout mice are susceptible to WD-induced liver injury, hepatic steatosis, apoptosis, inflammation, and fibrosis.	[89]
	(1) Mice fed with a HFD for 3 months; (2) 4-month-old Lep^ob/ob^ mice fed with a LFD	SESN2 knockout	No differ from control	Increased	Modestly increased	Increased	No data	Decreased	SESN2/AMPK-TSC2/mTOR/AKT	SESN2 knockout mice display aggravated hepatosteatosis, glucose intolerance, and insulin resistance.	[90]
	Mice fed with a HFD for 2 months	Adenoviral expression of SESN2	No differ from control	No data	Decreased	Decreased	No data	No data	SESN2/GATOR2-mTORC2/AKT	Adenoviral expression of SESN2 decreases lipogenesis and gluconeogenesis.	[91]
	Mice fed with a HFD for 2 months	Resveratrol administration	No data	Decreased	Decreased	No data	No data	No data	SESN2/LXRα-RXRα-LXRE/SREBP-1c	SESN2 induction by resveratrol contributes to the inhibition of the LXRα activity and lipogenesis.	[92]
	Mice fed with a MCD diet for 3 weeks	Carbon monoxide administration	No data	Decreased	No data	No data	Decreased ER stress and inflammation	Increased	PERK-eIF2α-ATF4/SESN2/AMPK/mTORC1	Carbon monoxide-enhanced SESN2 expression diminishes lipid accumulation and liver damage through autophagy.	[95]
	Mice fed with a HFD for 2 months	Liraglutide administration	Decreased	Decreased	No data	Decreased	Decreased oxidative stress and inflammation	No data	SESN2/Nrf2/HO-1	SESN2 induced by liraglutide mitigates hepatic lipid accumulation, oxidative stress, and inflammation.	[97]
AFLD	Mice received alcohol gavage for 4 weeks	Pterostilbene administration	Increased	Decreased	No data	No data	Decreased inflammation	Increased	SESN2/p62/CCN1	Induction of SESN2 by pterostilbene promotes p62-mediated autophagic degradation of CCN1 and relieves hepatic damage.	[100]

*AFLD* alcoholic fatty liver disease, *AKT* protein kinase B, *AMPK* adenosine monophosphate-activated protein kinase, *ATF4* activating transcription factor 4, *CCN1* cellular communication network factor 1, *eIF2α* alpha subunit of eukaryotic translation initiation factor 2, *ER* endoplasmic reticulum, *GATOR2* GTPase-activating protein activity towards Rags 2, *HFD* high-fat diet, *HO-1* heme-oxygenase-1, *JNK* c-Jun N-terminal kinase, *Keap1* Kelch-like ECH-associated protein 1, *LFD* low-fat diet, *LXRα* liver X receptor alpha, *LXRE* LXR response element, *MCD* methionine/choline deficiency, *mTORC1* mechanistic target of rapamycin complex 1, *NAFLD* non-alcoholic fatty liver disease, *Nrf2* nuclear factor E2-related factor 2, *PERK* protein kinase R-like endoplasmic reticulum kinase, *RXRα* retinoic acid receptor alpha, *SESN2* Sestrin2, *SREBP-1c* sterol regulatory element-binding protein 1c, *WD* Western diet.

### Hepatic fibrosis and cirrhosis

Hepatic fibrosis and cirrhosis are common advanced stages after chronic liver injuries and inflammatory response, including fatty liver diseases, viral hepatitis, etc. Fibrogenesis is featured by excessive extracellular matrix accumulation, which destroys intrahepatic structure, disrupts biological exchange of substances between hepatocytes and hepatic sinusoids, and is accompanied by extensive hepatocyte death, HSCs activation, and hypohepatia [101].

#### HSCs activation

SESN2 was firstly found to confer protection against hepatic fibrogenesis in obese mice and then in carbon tetrachloride (CCl_4_)- and bile duct ligation (BDL)-insulted fibrotic mice [60, 102, 103]. HSCs activation is the key event driving hepatic fibrogenesis. In primary HSCs isolated from murine livers that exposed to single dose of CCl_4_, SESN2 expression is markedly elevated. Concertedly, SESN2 is transcriptionally up-regulated in primary murine HSCs during in vitro auto-activation or in immortalized human HSCs line LX-2 cells stimulated by transforming growth factor-β (TGF-β) for 0–12 h [103]. Intriguingly, TGF-β induction for 48 h or long-term CCl_4_ damage for 8 weeks results in SESN2 reduction in rat HSC-T6 cells or murine livers [102]. Clinical liver specimens from advanced cirrhotic patients also show decreased hepatic SESN2 expression [103]. These findings suggested that SESN2 expression varies in different trends during early and advanced fibrotic responses and this may be valuable to the therapeutic discovery, but the molecular basis for this dynamic alternation needs further exploration. HSCs-specific delivery of SESN2 reduces α-SMA-labeled activated HSCs and collagen deposition, thereby ameliorating prolonged CCl_4_- or BDL-induced hepatic fibrosis in mice. Mechanistically, there has a possible interaction between SESN2 and TGF-β, a signal molecule that can activate adjacent quiescent HSCs, transform HSCs into myofibroblasts, and promote fibrosis development. TGF-β induction causes Smad3 phosphorylation and augments the binding of p-Smad3 to a putative Smad-binding element within *SESN2* gene promoter (-964 to -956 bp). In addition to Smad-dependent pathway, TGF-β-induced p38 MAPK activation and ROS production are also involved in SESN2 induction. SESN2 inhibits Smad3 phosphorylation but enhances Smad7 expression [102, 103]. Smad7 is a negative regulator of TGF-β/Smad pathway as Smad7 can bind to TGF-β receptor I (TGFβRI) and prevent the phosphorylation of Smad2 and Smad3, or recruit E3 ubiquitin ligase Smad ubiquitination regulatory factors (Smurfs) to Smad2 and TGFβRI and ubiquitinate and degrade the two proteins [104].

#### Inflammation

Liver inflammation is a key driver of HSCs activation and fibrogenesis. Hu et al. observed that lentiviral SESN2 overexpression abrogates CCl_4_-induced elevation of pro-inflammatory cytokines including TNF-α, IL-1β, and CCL2/MCP-1 in murine livers [102]. Yang et al. also found that recombinant adenovirus expressing SESN2 reduces CD45^+^ leukocytes in murine fibrotic livers caused by CCl_4_ or BDL [103]. Recently, Zhou et al. delineated that pharmacological induction of SESN2 decreases the number of CD45^+^ leukocytes and F4/80^+^ macrophages/Kupffer cells in murine livers under long-term alcohol exposure [100]. These findings imply that the anti-inflammatory action of SESN2 may be based on its modulation of inflammatory cell infiltration and activation in the liver.

In summary, current studies have preliminarily revealed the implication of SESN2 in ameliorating hepatic fibrosis (Table 2). However, fibrogenesis involves multiple types of hepatic cells including HSCs, hepatocytes, Kupffer cells, liver sinusoidal endothelial cell, etc., thus, whether and how SESN2 in other types of hepatic cells influences the process of hepatic fibrosis remain inconclusive.

**Table 2:** Roles of SESN2 in hepatic fibrosis and cirrhosis.

Study subjects/Model		SESN2 expression		Intervention	HSCs activation and α-SMA expression	Overall outcome	Reference
		mRNA	protein				
In vitro models	Primary HSCs isolated from CCl_4_‐treated mice	No data	Increased	No data	No data	No data	[103]
	Auto-activated primary HSCs from healthy wild-type mice	Increased	Increased	No data	No data	No data	[103]
	LX-2 cells treated with TGF-β for no more than 12 h	Increased	Increased	Plasmid-mediated SESN2 overexpression	Decreased	No data	[103]
	HSC-T6 cells treated with TGF-β for 48 h	Decreased	Decreased	Plasmid-mediated SESN2 overexpression	Decreased	No data	[102]
In vivo models	Mice fed with HFD for induction of non-alcoholic fatty liver fibrosis	No data	Increased	SESN2 knockout	Increased	SESN2 ablation provokes HSCs activation, collagen production, and hepatic fibrogenesis.	[60]
	Mice injected with CCl_4_ or received BDL for induction of hepatic fibrosis	No data	Decreased	Adenoviral or lentiviral expression of SESN2	Decreased	Exogenous SESN2 expression contributes to decreased serum ALT and AST activities, inflammatory cell infiltration, and hepatic collagen deposition.	[102, 103]
Clinical samples	Cirrhotic liver tissues	Decreased	Decreased	No data	No data	SESN2 expression is decreased in cirrhotic livers and negatively correlated with disease progression.	[103]

*ALT* alanine aminotransferase, *AST* aspartate aminotransferase, *α-SMA* alpha smooth muscle actin, *BDL* bile duct ligation, *CCl*_*4*_ carbon tetrachloride, *HFD* high-fat diet, *HSCs* hepatic stellate cells, *SESN2* Sestrin2, *TGF-β* transforming growth factor beta.

### Liver cancer

Liver cancer has the sixth highest incidence and the fourth highest mortality rate among cancers worldwide, which can be derived from chronic liver diseases, etc. HCC accounts for approximately 90% of liver cancer cases [105]. Chen et al. and Qi et al. reported that SESN2 expression is dramatically lower in HCC tissues than that in adjacent non-cancerous tissues, which is highly correlated with lymph node metastasis, tumor progression, and poor prognosis in HCC patients [106, 107]. However, Dai et al. disputed that SESN2 abundance is higher in HCC tissues than that in corresponding adjacent non-cancerous liver tissues. Coherently, SESN2 levels are higher in HCC cell lines, including Bel-7404, SNU-368, HLE, HLF, and Hep3B cells, comparing with normal human hepatocytes HL-7702 cells [108]. The contradictory findings between the studies may be owing to the discrepancy and insufficiency of HCC samples or the comorbidities in HCC patients, which needs more comprehensive explorations.

#### Autophagy

SESN2 has been well-documented in initiating autophagy, however, the role of autophagy machinery in HCC is paradoxical, so is SESN2. Wang et al. found that fangchinoline induces autophagic death of hepatoma cell lines HepG2 and PLC/PRF/5 cells via activating p53/SESN2/AMPK signaling [109]. Qi et al. confirmed that SESN2/AMPK/mTOR1 signaling induced by muscone triggers autophagy-dependent apoptosis of HepG2 cells [107]. The researches highlight the anti-oncogenic effect of SESN2 and autophagy. However, autophagy is defined as a double-edged sword as it can be beneficial to cancer cells by preventing oxidative stress, DNA damage, and inflammation, or starvation [110]. Jegal et al. found that SESN2-dependent autophagy induced by eupatilin protects HepG2 cells from arachidonic acid and iron-induced oxidative stress and promotes cell survival [41]. The heterophany of SESN2-dependent autophagy in HCC may be associated with the difference in metabolic environments of HCC cells or drug administration that may activate unrevealed signaling cascades.

#### Cell survival and death

Induction of apoptotic cell death can restrain the proliferation, invasion, and migration of HCC cells, which may be a viable anti-cancer strategy [111, 112]. Several studies have confirmed that SESN2 can promote apoptosis of multiple types of cancer cells, including human head and neck cancer cells, lung adenocarcinoma cells, and colon cancer cells [113–115]. However, intriguingly, up-regulated SESN2 in HepG2 cells halts cell apoptosis and exacerbates primary resistance to sorafenib, which is attributed to activation of pro-survival AKT and AMPK signaling pathways [108]. Kumar et al. found that SESN2 forms a complex with JNK and FoxO1 and promotes FoxO1 nuclear translocation, elevating the transcriptional level of peroxisome proliferator-activated receptor γ coactivator 1α (PGC-1α). PGC-1α can promote glutamine metabolism, increase mitochondrial biogenesis, and decrease the expression of pro-apoptotic genes, such as p53 up-regulated modulator of apoptosis (PUMA) and B-cell lymphoma-2-associated X protein (Bax), facilitating the survival of HepG2 cells under glucose starvation conditions [116]. The function of SESN2 in different cancer cells may depend on the species or cellular metabolic conditions.

Altogether, SESN2 has been preliminarily shown to regulate autophagy and cell status in HCC (Table 3), but its concrete effects are far from clear. In vivo studies are needed to further determinate the association between SESN2 and HCC pathology, including tumor size and number, tumor stage, survival rate, prognosis, etc., and testify the therapeutic implication of SESN2 modulation.

**Table 3:** Roles of SESN2 in HCC.

Study subjects/Model	SESN2 expression	Major conclusion	Mechanism	Reference
HCC cell lines, including HepG2, H4IIE, and Hepa-1c1c7 cells	The levels of SESN2 mRNA and protein are up-regulated by eupatilin in HCC cells.	SESN2 induced by eupatilin protects HCC cells from oxidative stress.	Promote autophagy	[41]
Fresh HCC tissues (*n* = 15) and paired non-cancerous tissues (*n* = 15)	The levels of SESN2 mRNA and protein are statistically lower in HCC tissues than that in non-cancerous tissues.	SESN2 level is lower in HCC tissues, which is correlated with hepatitis B/C viral infections, lymph node metastasis, and tumor progression. High expression of SESN2 implies advantageous prognosis in HCC patients.	No data	[106]
Fixed and embedded HCC tissues (*n* = 100) and paired non-cancerous tissue samples (n = 100)	38% (38/100) HCC tissues display high SESN2 expression.			
	71% (71/100) non-cancerous tissues display high SESN2 expression.			
Fresh HCC tissues (*n* = 14) and corresponding non-cancerous tissues (*n* = 14)	The levels of SESN2 mRNA and protein are lower in HCC tissues than that in non-cancerous tissues.	SESN2 level is up-regulated by muscone and inhibits tumor growth.	Promote SESN2/AMPK/mTORC1-dependent autophagy and apoptosis	[107]
HepG2 cells xenograft tumors grown in BALB/c nude mice	The levels of SESN2 mRNA and protein are up-regulated by muscone in HepG2 cell subcutaneous tumors and HepG2 cells.			
HCC cell line HepG2 cells				
HCC tissues and corresponding adjacent non-cancerous tissues (*n* = 30)	SESN2 expression is up‐regulated in HCC tissues when compared with adjacent non-cancerous tissues.	SESN2 level is elevated in HCC tissues and cells, which promotes cell proliferation, facilitates sorafenib primary resistance, and inhibits cell apoptosis.	Activate AKT and AMPK signaling pathways	[108]
HCC cell lines, including Bel‐7404, HLF, HLE, SNU‐368, and Hep3B cells	The levels of SESN2 mRNA and protein are higher in HCC cell lines than that in normal human hepatocyte line HL-7702 cells.			
HCC cell lines, including HepG2 and PLC/PRF/5 cells	The level of SESN2 mRNA is up-regulated by fangchinoline in HCC cells.	SESN2 induced by fangchinoline promotes autophagic cell death in HCC cells.	Activate p53/SESN2/AMPK signaling	[109]
HCC cell line HepG2 cells	SESN2 expression is up-regulated under glucose-deprived conditions.	SESN2 promotes intracellular glutamine metabolism and cell survival under glucose starvation in HepG2 cells.	Promote SESN2/JNK/FoxO1/PGC-1α activation	[116]

*AKT* protein kinase B, *AMPK* adenosine monophosphate-activated protein kinase, *FoxO1* forkhead box protein O1, *HCC* hepatocellular carcinoma, *JNK* c-Jun N-terminal kinase, *mTORC1* mechanistic target of rapamycin complex 1, *PGC-1α* peroxisome proliferator-activated receptor γ coactivator 1α, *SESN2* Sestrin2.

## Conclusion and future directions

In this review, we summarize that SESN2 regulates multiple cellular events including glycolipid metabolism, oxidative stress, ER stress, HSCs activation, inflammation, autophagy, cell survival and death and integrate the multicomponent network involved in SESN2 action. From preclinical studies, we also conclude that SESN2 is involved in the development and progression of acute and chronic liver diseases and serve as an endogenous hepatoprotective molecule.

Evidence has shown that SESN2 is of great clinical significance in a variety of diseases. Circulating SESN2 levels have been identified viable in indicating disease severity or prognosis, including cardiovascular diseases [117–119], respiratory diseases [120, 121], neurodegenerative diseases [122, 123], metabolic diseases [124, 125], cancers [106, 126], etc. Since SESN2 expression is dynamically changed during liver pathology, we hypothesize that SESN2 has a good potential as a clinical biomarker and prognostic indicator for liver diseases. In addition, the findings from preclinical studies uncover the favorable outcome of SESN2 regulation in the intervention of liver damage with no obvious side effects, which suggests that SESN2 may be a promising therapeutic target for liver diseases. Future work focusing on identifying compounds that induce or activate SESN2 may drive the development of hepatoprotective strategies. Moreover, direct targeting at SESN2 using genetical techniques like viral vector delivery system may be tested in future clinical trials.
